# Recycling implants: a sustainable solution for musculoskeletal research

**DOI:** 10.1080/17453674.2019.1706301

**Published:** 2020-01-06

**Authors:** Lars Lidgren, Deepak Bushan Raina, Magnus Tägil, K Elizabeth Tanner

**Affiliations:** a Lund University, Faculty of Medicine, Department of Clinical Sciences Lund, Orthopedics, Lund, Sweden;; b School of Engineering and Materials Science, Queen Mary University of London, UK

About half a million people in Sweden (5%) (10.5 million inhabitants) walk around with a bone or joint implant. This could be extrapolated to 50 million only in the Americas, Europe, and the Pacific. Orthopedic surgeons are running the largest workshop for repairing humans worldwide, and, in fact, one-fifth of all people older than 75 years in Sweden have an artificial joint. In the United States, 5–10% of all hip or knee implants are being revised during a lifetime (Malchau et al. 2018).

The environmental as well the financial aspects of recycling metals at revision or post-mortem has thus far not been prioritized on the orthopedic societies’ agendas.

Close to 100,000 Swedes pass away annually, with 70% being cremated. To protect the environment, the Swedish Government changed its regulation in 2016, making it mandatory for the Swedish Church—which is responsible for all funerals—to arrange for recycling after cremations of all metal components including the coffin. Since 2016, all metal collected at cremation has been recycled (Figure), resulting in 60 tons of valuable metals (such as, for instance, titanium) at a net value of US$15 million. The recycling company, however, charges 20%, while 80% is plowed back to support societal projects handled by a large general inheritance fund (Hyckenberg 2019). If the Swedish figures were extrapolated to Europe and the United States alone, the total income from metal recycling at funeral would be around US$250 million annually (i.e., 700 million inhabitants, assuming 1% mortality per year, 50% cremation and if 20% had a metal device at death) (The Cremation Society 2019).

In addition, recycling of extracted implants that are currently scrapped as dangerous goods should be possible if the logistics for collection could be handled properly. This could be done under the auspices of the national orthopedic societies. With an expected minimal cumulative revision rate of 5% leading to a revision arthroplasty, this recycling in Sweden should give a yearly recurring income close to US$500,000, which could be directed to orthopedic research. Given the ongoing “age quake” and an increasing cremation trend in the industrialized world, metal implant recycling—which has just started in Sweden—is likely to spread to other countries.

Leading implant manufacturers are encouraged to start collaborating with the orthopedic societies, who should assume their environmental responsibility and facilitate recycling of metal implants both at revision and at post-mortem. This has to be done in a manner respectful to the deceased and his or her loved ones.

In cooperation with patient organizations, we suggest that orthopedic societies put forward a motion to governments that at least part of the income from such recycling be directed to musculoskeletal research in the respective countries.
